# Treatment with idelalisib in patients with chronic lymphocytic leukemia – real world data from the registry of the German CLL Study Group

**DOI:** 10.1007/s00277-023-05314-2

**Published:** 2023-06-26

**Authors:** Julia von Tresckow, Nikola Heyl, Sandra Robrecht, Adam Giza, Ali Aldaoud, Rudolf Schlag, Martine Klausmann, Hartmut Linde, Wolfgang Stein, Andreas Schwarzer, Kirsten Fischer, Paula Cramer, Barbara Eichhorst, Michael Hallek, Anna Maria Fink

**Affiliations:** 1 Clinic for Hematology and Stem Cell Transplantation, West German Cancer Center, University Hospital Essen, University of Duisburg-Essen, Hufelandstr. 55, 45147 Essen, Germany; 2 Department I for Internal Medicine and Centre of Integrated Oncology Aachen, Bonn, Cologne, Duesseldorf, Faculty of Medicine and University Hospital Cologne, University of Cologne, Cologne, Germany; 3 Praxis für Hämatologie und Onkologie, Leipzig, Germany; 4 Hämatologisch-Onkologische Schwerpunktpraxis, Würzburg, Germany; 5 Gemeinschaftspraxis, Aschaffenburg, Aschaffenburg, Germany; 6 MVZ für Blut und Krebserkrankungen, Potsdam, Germany; 7 Klinikum Frankfurt Oder, Frankfurt, Germany; 8 Onkopraxis Probstheida, Leipzig, Germany

**Keywords:** Idelalisib, Chronic lymphocytic leukemia, Real-world-data

## Abstract

Idelalisib in combination with rituximab is an efficacious treatment for patients suffering from chronic lymphocytic leukemia (CLL) with known limitations due to toxicities. However, the benefit after prior Bruton tyrosine kinase inhibitor (BTKi) therapy remains unclear. For this analysis, 81 patients included in a non-interventional registry study of the German CLL study group (registered at www.clinicaltrials.gov as # NCT02863692) meeting the predefined criteria of a confirmed diagnosis of CLL and being treated with idelalisib containing regimens outside clinical trials were considered. 11 patients were treatment naïve (13.6%) and 70 patients (86.4%) pretreated. Patients had median of one prior therapy line (range 0–11). Median treatment duration with idelalisib was 5.1 months (range 0–55.0 months). Of 58 patients with documented treatment outcome, 39 responded to idelalisib containing therapy (67.2%). Patients treated with the BTKi ibrutinib as last prior treatment prior to idelalisib responded in 71.4% compared to a response rate of 61.9% in patients without prior ibrutinib. Median event free survival (EFS) was 15.9 months with a 16 versus 14 months EFS in patients with ibrutinib as last prior treatment or not, respectively. Median overall survival was 46.6 months. In conclusion, treatment with idelalisib appears to have a valuable impact in patients being refractory to prior ibrutinib therapy even though there are limitations in our analysis due to the low number of patients included.

## Introduction

The oral first-in-class phosphatidylinositol 3-kinase delta (PI3Kδ) inhibitor idelalisib in combination with the CD20-antibody rituximab has been approved for therapy of chronic lymphocytic leukaemia (CLL) back in 2014 and is indicated for the treatment of patients with at least one prior therapy or first-line treatment in presence of *deletion 17p (del17p)* or *TP53* mutation in patients who are not eligible for any other drugs. In 2016, serious side effects and an increased fatality rate were documented in ongoing clinical trials, so that further safety measurements were implemented and application in first-line therapy was restricted.

Efficacy of idelalisib in combination with rituximab is proven in heavily pre-treated patients with CLL [1, 2]. Even in routine practice, a clear efficacy of the drug was described [3].

However, it remains unclear which patients may benefit the most from the combination and how the drug is rated in different treatment lines. Furthermore, the benefit of idelalisib containing therapy is unclear after prior treatment with the Bruton Tyrosin Kinase Inhibitor (BTKi) ibrutinib.

## Methods

In this non-interventional registry study of the German CLL Study Group (GCLLSG) all patients with a confirmed CLL, irrespective of treatment regimens as well as therapy line including watch and wait patients, can be included and are followed until death. Patients were managed in outpatient hematological departments, peripheral hospitals as well as in academic centers. Data on status of the disease and treatment if applicable (including duration of treatment) were collected at least annually. Patient demographics, including cytogenetic aberrations and IGHV gene status, were collected at time of first diagnosis and start of each line of treatment. The primary endpoint of the registry is overall survival (OS), secondary endpoints include event-free survival (EFS) and time to next treatment (TTNT), overall response rate (ORR) of all treatment lines, secondary diseases such as secondary malignancies, autoimmune diseases and infections, course of the disease in predefined biological subgroups as well as the quality of life. The registry was approved by all involved Ethic committees according to the declaration of Helsinki and German physicians’ professional regulation. All patients provided informed consent. The registry is registered at www.clinicaltrials.gov as #NCT02863692.

This analysis considers all patients included in the above mentioned GCLLSG registry study meeting the predefined criteria of a confirmed diagnosis of CLL and being treated at least one time with idelalisib containing regimens (defined as treatment with idelalisib/rituximab and/or idelalisib/ofatumumab); however, patients participating in a clinical trial of the GCLLSG evaluating idelalisib (CLL2-BCG, CLLR-Umbrella1, CLLR-Umbrella2 [4]) were excluded.

### Statistical analysis

Patient, CLL and treatment characteristics including ORR and treatment duration, which was defined as the time between start and stop date of treatment (except for ongoing therapies, where the last date of observation was used as stop date), were reported using counts and percentages for categorical variables and descriptive statistics for continuous variables. For demographics and CLL characteristics, values at start of first idelalisib treatment were used.

EFS was defined as the interval between start of treatment and disease progression, start of next treatment or death of any cause. TTNT was calculated from start of treatment to start of next therapy and OS from start of therapy to death.

Time-to-event endpoints were analyzed from start of last therapy prior first idelalisib therapy, from start of first idelalisib treatment, and from start of first subsequent treatment after first idelalisib therapy using Kaplan–Meier methods.

Analyses were done using SPSS version 26.

## Results

### Patients

For this analysis, 4994 patients that were included at the timepoint of June 2020 in the registry of the GCLLSG were considered. 81 patients from 36 sites in Germany met the predefined criteria and were included in the analysis population of this study. 11 patients were still on first idelalisib containing therapy at the timepoint of the data cut-off. The median observation time from start of first-line therapy was 109.7 months (range 2.6–254.4 months).

Patient characteristics are shown in Table 1.

**Table 1:** Patient characteristics. Table 1 shows the characteristics of the patients considered for this analysis at the time point of the first idelalisib containing regimen

Characteristic	Total
Age	81
Median (range)	74 (51–94)
≤ 65 years, *N* (%)	17 (21.0)
> 65 years, *N* (%)	64 (79.0)
Gender	81
Female, *N* (%)	25 (30.9)
Male, *N* (%)	56 (69.1)
CIRS score	63
Median (range)	3 (0–17)
≤ 6, *N* (%)	47 (74.6)
> 6, *N* (%)	16 (25.4)
Missing information, *N* (%)	18 (22.2)
Creatinine clearance (ml/min)	49
< 70, *N* (%)	28 (57.1)
≥ 70, *N* (%)	21 (42.9)
Missing information, N (%)	32 (39.5)
ECOG performance status	40
0, *N* (%)	18 (45.0)
1, *N* (%)	18 (45.0)
2, *N* (%)	4 (10.0)
Missing information, N (%)	41 (50.6)
Binet stage	55
A, *N* (%)	7 (12.7)
B, *N* (%)	28 (50.9)
C, *N* (%)	20 (36.4)
Missing information, *N* (%)	26 (32.1)
IGHV mutational status	13
Unmutated, *N* (%)	9 (69.2)
Mutated, *N* (%)	4 (30.8)
Missing information, *N* (%)	68 (84.0)
*TP53*mutational status	13
Unmutated, *N* (%)	7 (53.8)
Mutated, *N* (%)	6 (46.2)
Missing information, *N* (%)	68 (84.0)
*TP53* status	16
No *del17p* or *TP53* mutation, *N* (%)	7 (43.8)
* Del17p* and/or *TP53* mutation, *N* (%)	9 (56.3)
Missing information, *N* (%)	65 (80.2)
Cytogenetic subgroups by hierarchical order	19
* Del17p*, *N* (%)	4 (21.1)
* Del11q*, *N* (%)	1 (5.3)
Trisomy 12, *N* (%)	4 (21.1)
No abnormalities, *N* (%)	7 (36.8)
* Del13q* only, *N* (%)	3 (15.8)
Missing information, *N* (%)	62 (76.5)

### Idelalisib containing therapy

**Fig. 1:**
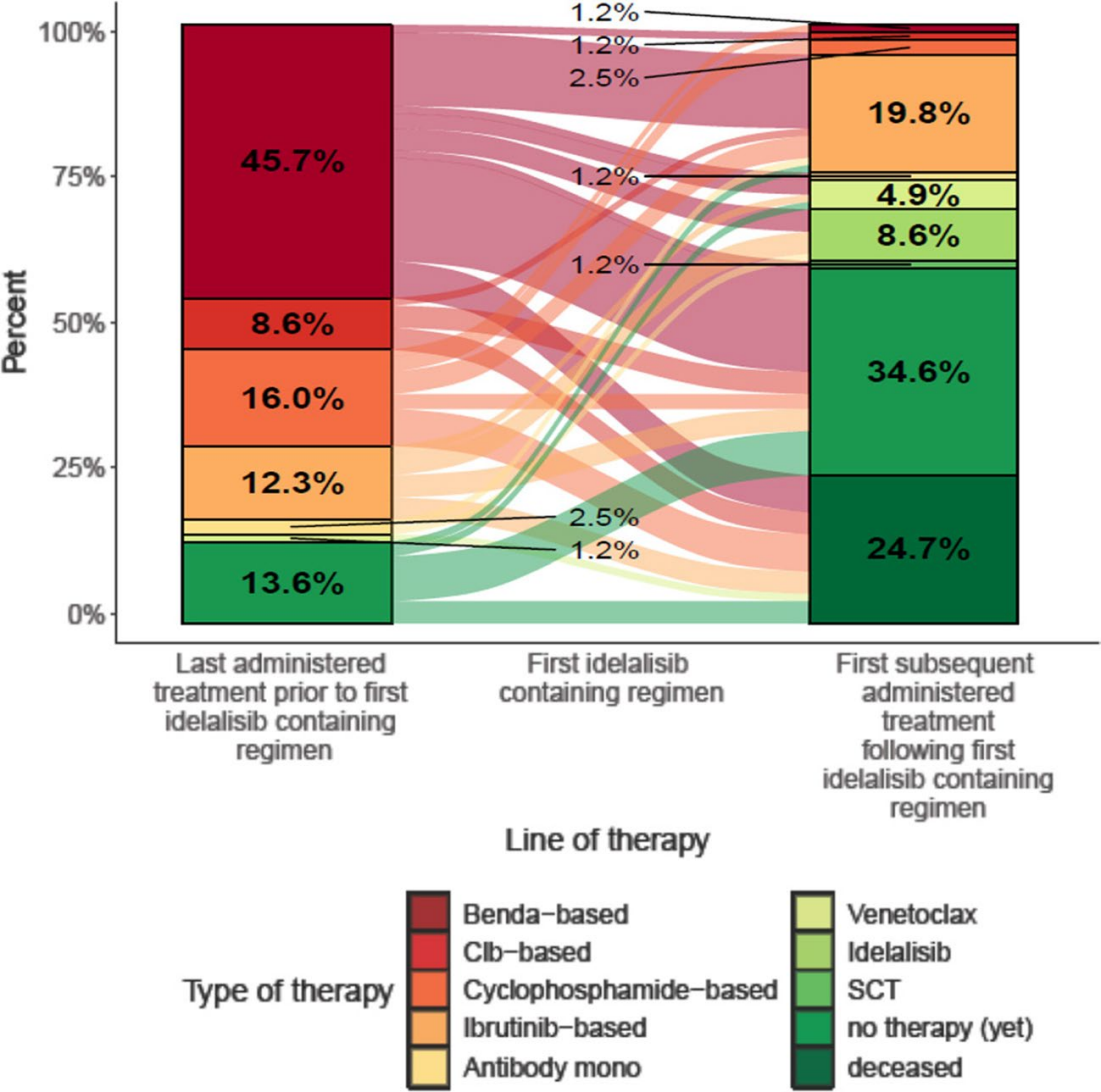
Therapy sequence. The Sankey plot in Fig. 1 shows the last administered treatment prior to first idelalisib containing regimen as well as the first administered treatment following first idelalisib containing regimen. Benda = Bendamustin Clb = Chlorambucil SCT = stem cell transplantation

The median time from first diagnosis until the start of first idelalisib containing therapy was 80.5 months (range 1.2–278.3 months). All 81 patients received idelalisib in combination with rituximab. Treatment was most frequently (25 patients, 30.9%) started in 2015 (before the dear doctor letter on idelalisib was issued by the European medical agency). However, one patient started therapy in 2013 (1.2%), 13 patients in 2014 (16.0%), 16 patients in 2016 (19.8%), 13 patients in 2017 (16.0%), 11 patients in 2018 (13.6%) and 2 patients in 2019 (2.5%), respectively. 70 patients received prior CLL therapy before administration of idelalisib whereas 33 patients received further therapy after termination of idelalisib treatment. Therapy sequence is shown in Fig. 1.

### Prior therapies

A median of one prior therapy was administered before start of the idelalisib containing therapy (range 0–11). Eleven patients received idelalisib as first-line therapy (13.6%), 32 patients as second-line (39.5%) and 14 patients as third-line treatment (17.3%). Twenty-four patients received ≥ 3 prior therapies (29.6%).

With 46.9% of all administered treatment regimens, bendamustin containing therapies were most commonly given as prior therapy. Venetoclax constituted 1.1% of the prior therapies whereas ibrutinib containing regimens were administered in 7.4%.

Fifty-nine of 70 pretreated patients (84.3%) did not receive ibrutinib before start of idelalisib therapy. One patient was treated with ibrutinib as any other prior therapy (1.4%) whereas 10 patients had ibrutinib as the last prior treatment before idelalisib (14.3%).

### Outcome of first line treatment

Eleven patients (13.6%) received idelalisib plus rituximab as first-line treatment, 1 patient ibrutinib (1.2%) and 69 patients (85.2%) chemotherapy with or without rituximab. As chemoimmunotherapy, bendamustin plus rituximab was most commonly given in 32 patients (39.5%). The median EFS from start of first-line treatment was 31.7 months with a one-year survival of 82.6%, a 3-year-survival of 42.6% and a 5-year-survival of 13.8%. Progressive disease (PD) after start of first-line therapy is documented in 58 patients of the analysis population. In 32 patients (55.2%), the time between start of first-line treatment and PD was ≤ 36 months and in 26 patients (44.8%) > 36 months.

### Last treatment prior to first idelalisib containing regimen

Response to last treatment prior to first idelalisib therapy summarized in Table 2.

**Table 2:** Summarizes data on previous and subsequent therapies

Previous and subsequent treatment	81
Patients with administered treatments prior to first idelalisib containing regimen, *N* (%)	70 (86.4)
Patients with administered treatments after first idelalisib containing regimen, *N* (%)	33 (40.7)
Number of administered treatments prior to first idelalisib containing regimen, *N* (%)	81
≤ 1, *N* (%)	43 (53.1)
2, *N* (%)	14 (17.3)
≥ 3, *N* (%)	24 (29.6)
Response to last treatment prior to first idelalisib containing regimen	57
Response, *N* (%)	41 (71.9)
No response, *N* (%)	16 (28.1)
Missing information, *N* (%)	13 (18.6)
Response to ibrutinib as last treatment prior to first idelalisib containing regimen	7
Response, *N* (%)	3 (42.9)
No response, *N* (%)	4 (57.1)
Missing information, *N* (%)	3 (30.0)
Response to non-ibrutinib containing therapy as prior treatment to first idelalisib containing regimen	49
Response, *N* (%)	38 (77.6)
No response, *N* (%)	11 (22.4)
Missing information, *N* (%)	10 (16.9)

Forty-one of 57 patients with documented response assessment for last treatment prior to idelalisib therapy responded (71.9%) and 16 patients did not respond (28.1%).

The median time to first idelalisib containing therapy from start of last prior treatment was 22.3 months. For patients with ibrutinib as last prior treatment, median time was 8.1 months, for the patient with ibrutinib as any other prior treatment 5.7 months and for patients without prior ibrutinib therapy 29.0 months.

The median EFS form start of last treatment prior to first idelalisib containing therapy was 19.6 months. For patients with ibrutinib as last prior treatment median EFS was 8.4 months, for the patient with ibrutinib as any other prior treatment 5.7 months and for patients without prior ibrutinib therapy 27.4 months.

Median OS from start of last treatment prior to first idelalisib containing therapy was not reached with a 3-year survival of 80.7%. For patients with ibrutinib as last prior treatment median OS was not reached with a 3-year survival of 77.8%. For the patient with ibrutinib as any other prior treatment OS was 9.2 months whereas the median OS for patients without prior ibrutinib therapy was not reached with a 3-year survival of 82.5%.

### Treatment duration of first idelalisib containing regimen

Median treatment duration with first idelalisib containing regimen was 5.1 months (range 0.0—55.0 months), while the median treatment duration was 2.8 month (range 1.0–36.1 months) in patients with idelalisib containing regimen as first-line therapy.

### Response to first idelalisib containing regimen

For 58 patients, information about treatment outcome is available; 39 patients (67.2%) responded, 19 patients did not respond (32.9%). Information about treatment outcome is missing in 23 patients (28.4%). Seven of 11 patients who received idelalisib plus rituximab as first-line therapy responded. Information is missing for 4 patients.

Five of 7 patients with ibrutinib as last prior therapy responded to idelalisib (71.4%). Information is missing for 3 patients. In contrast, the patient with ibrutinib as any other prior therapy did not respond and 26 of 42 patients without any prior ibrutinib therapy responded (61.9%). Information is missing in 17 patients.

Furthermore, all patients with *del17p* (4 patients, 100.0%) and all patients with *TP53* mutation (5 patients, 100.0%) responded. For the latter, information is missing in 1 patient. In contrast, 9 of 12 patients without *del17p* (75.0%) and 6 of 7 patients without *TP53* mutation (85.7%) responded. Information is missing in 4 and 0 patients, respectively. Taking these high-risk features together again, all patients with *del17p* and/or *TP53* mutation responded (8 patients, 100.0%). Information is missing in 1 patient. Concerning 7 patients with neither *del17p* nor *TP53* mutation, 6 patients responded (85.7%).

With regard to the number of prior treatment lines, 29 of 33 patients with ≤ 1 prior treatment (87.9%) responded whereas the response rate was 20% in 10 patients with 2 prior treatment lines and 53.3% in 15 patients with ≥ 3 prior therapies. Information is missing in 10, 4 and 9 patients, respectively.

### Time to next treatment (TTNT)

**Fig. 2:**
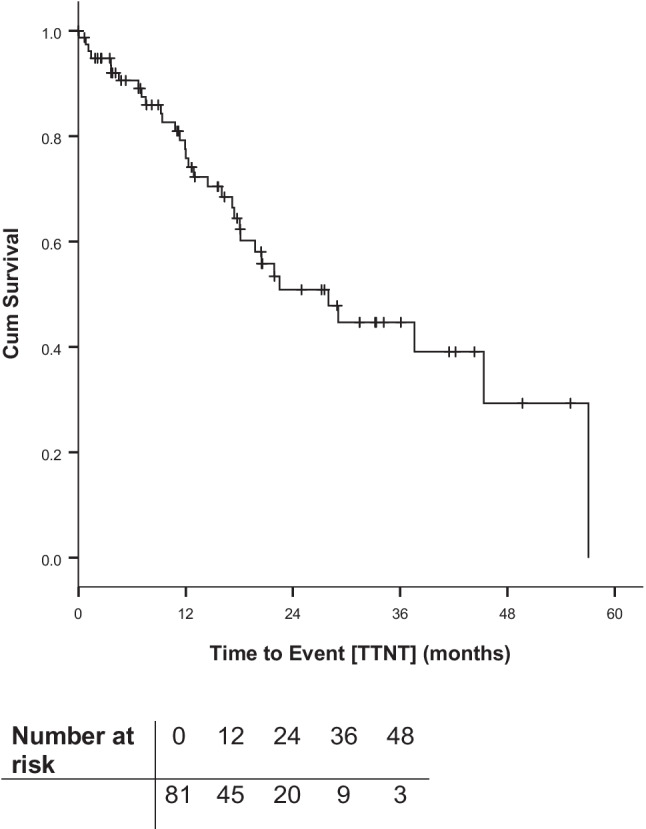
Time to next treatment (TTNT) from start of first idelalisib containing regimen

TTNT is shown in Fig. 2.

Median TTNT was 28.0 months after start of first idelalisib containing regimen with a 6-months survival of 90.6%, a 12-months survival of 77.5% and a 24-months-survival of 50.9%.

According to the number of administered treatments prior to idelalisib therapy, median TTNT was 29.0 months in patients with ≤ 1 treatment, 12.9 months in patients with 2 treatments and 28.0 months in patients with ≥ 3 treatments.

### Event free survival (EFS)

**Fig. 3:**
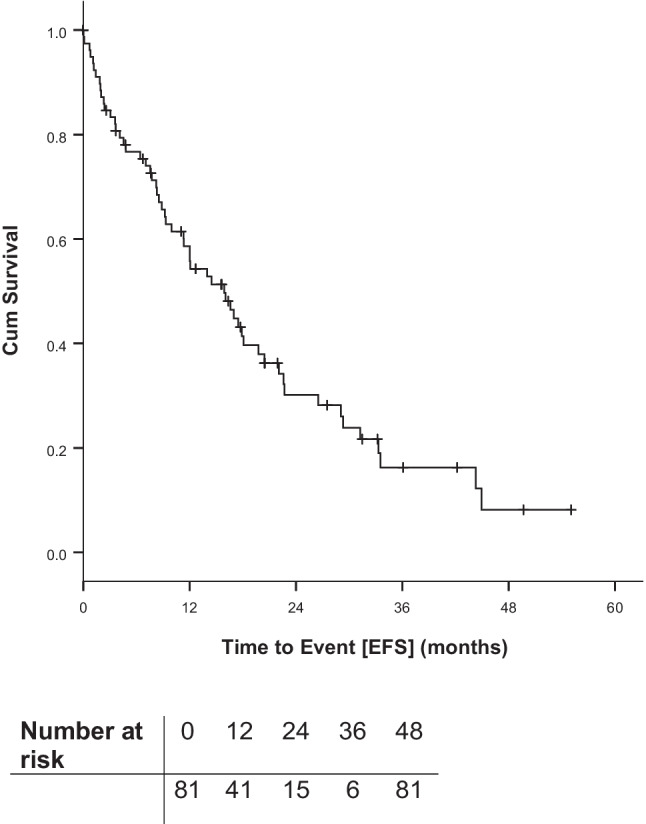
Event-free survival (EFS) from start of first idelalisib containing regimen

EFS is shown in Fig. 3.

Median EFS was 15.9 months after start of first idelalisib containing regimen with a 6-months survival of 76.7%, a 12-months survival of 58.6% and a 24-months survival of 30.2%.

### Overall survival

**Fig. 4:**
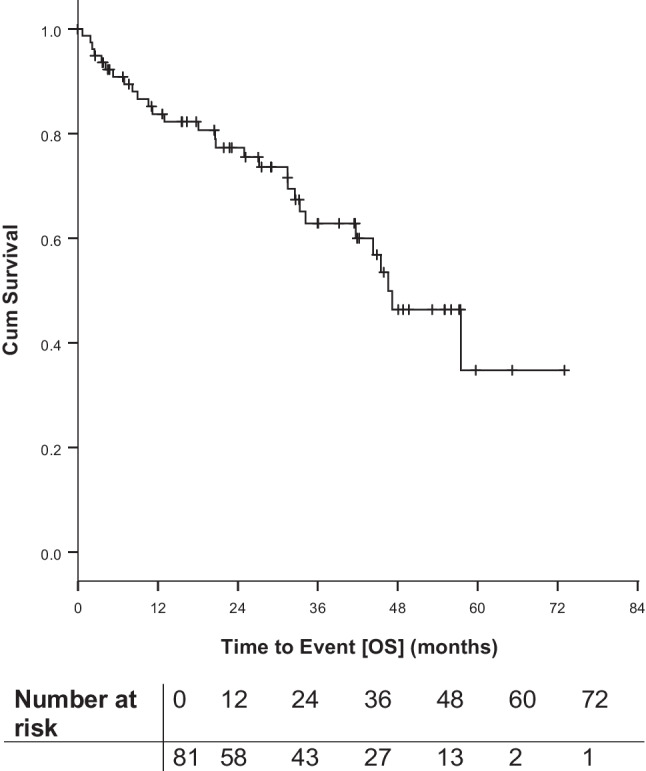
Overall survival (OS) from start of first idelalisib containing regimen

OS is shown in Fig. 4.

29 of 81 patients (35.8%) died. Causes of death included 10 PD (34.5%) including two patients with Richter’s transformation (RT) and one patient whose death was additionally caused by progressive myelodysplastic syndrome. One fatality (3.4%) was a pneumonia and deemed as treatment related. Besides this, 4 cases of infections (13.8%) including 2 cases of pneumonia, 2 cases of concomitant diseases (6.9%; cardiac insufficiency, decompensation of liver cirrhosis,) and 4 cases of other malignancies (13.8%; metastatic bronchial-carcinoma, metastatic urothelial carcinoma, squamous cell carcinoma, esophagus-carcinoma) were documented as causes of death. For 8 patients the reason for death is unknown.

Median OS was 46.6 months after start of first idelalisib containing regimen with a 6-months survival of 90.9%, a 12-months survival of 83.8% and a 24-months survival of 77.3%.

### Richter´s Transformation (RT)

Three patients (3.7%) achieved RT after start of first idelalisib containing regimen. These patients were pretreated with chemoimmunotherapy before administration of idelalisib.

### Subsequent treatment after idelalisib containing therapy

Fifty-six subsequent treatment regimens were administered in 33 patients after first idelalisib therapy. Ibrutinib- (37.5%) and venetoclax-containing regimes (21.4%) were administered most frequently. Stem cell transplant was performed in 7.1%. Further therapies consisted of chemo- or chemoimmunotherapy. Additionally, ibrutinib-containing regimens were the most frequent first subsequent therapy after idelalisib containing therapy (48.5%). For 20 patients the response of the first subsequent therapy is documented with 10 patients (50%) responding.

48 patients did not receive subsequent therapy after first idelalisib containing regimen. Of these, 20 patients died (41.7%). Eight patients died due to progressive disease (40.0%), including two patients with RT One death was a pneumonia and deemed as treatment related (5.0%). Three death cases were related to infections (15.0%), including 2 patients with pneumonia, and 4 patients died due to other malignancies (20.0%), including one patient with metastatic bronchial, metastatic urothelial carcinoma, squamous cell carcinoma, and oesophagus carcinoma each. For 4 patients the cause of death is not known.

28 patients without subsequent therapy after first idelalisib containing regimen were still alive. Of these, the median treatment duration in 17 patients with discontinued idelalisib therapy was 5.1 months (range 1.7–17.9 months) whereas the median treatment duration in 11 patients with ongoing idelalisib therapy was 12.7 months (range 0.0–55.0 months).

Median EFS after start of first subsequent treatment following first idelalisib containing regimen was 10.7 months with a 6-months survival of 75.0%, a 12-months survival of 47.5% and a 24-months survival of 38.4%.

Median OS after start of first subsequent treatment following first idelalisib containing regimen was 46.7 months with a 6-months survival of 100.0%, a 12-months survival of 92.3% and a 24-months survival of 78.4%.

## Discussion

With this real-world analysis of patients being documented in the registry of the GCLLSG we aimed to evaluate the outcome of idelalisib containing therapies in patients with CLL, especially after prior treatment with the BTKi ibrutinib.

With an ORR of 67.2% and a 12-months OS of 83.8% our data seem inferior compared to what was described in clinical trials testing idelalisib plus rituximab with an ORR of 81% and a 12-months OS of 92% [1], even though these data were documented in patients with relapsed/refractory CLL only. Certainly, it must be considered that data from real-world analyses are difficult to compare with results generated by randomized clinical trials.

As only progression-free survival (PFS) rather than TTNT or EFS is described in other real-world studies including idelalisib, our data are also difficult to compare to these studies. However, response rates are lower and OS seem to be shorter in our analysis [5, 6], even in comparison with real-world data evaluating ibrutinib- or venetoclax-based therapies [5, 7, 8]. This underlines the restricted benefit of idelalisib application in treatment of CLL that is already evident in clinical practice.

Though, we wanted to raise the question if there is still a value for using idelalisib in the progressing treatment landscape of CLL. As the declining use of the drug over the years shows, the application of idelalisib is already small. However, there might be a group of patients that benefit from treatment with idelalisib containing regimes. With a response rate of 42.9% and a median TTNT of only 8.1 months in patients with ibrutinib as last prior treatment prior to idelalisib, it must be assumed that these patients were almost refractory to treatment. Notably, 71.4% of these patients responded to idelalisib with a median EFS of 16.0 months. This underlines the potential value of the drug as bridging therapy to definite therapies such as CAR-T-therapy in the future or allogeneic stem cell transplant for eligible patients.

The main limitation of our analysis is the small number of patients that were included. Hence, especially the validity of subgroup analyses is limited. Furthermore, we neither can conclude on the reason for the relatively short treatment duration nor on the type of adverse events that probably led to treatment discontinuation, because these data are not collected in the German CLL study group registry. The question, whether there is a place for idelalisib or other PI3K inhibitors in CLL treatment, particularly in patients who were refractory to BTK inhibitors or venetoclax, cannot be answered with our data, because most of the patients were not treated with targeted agents but with alkylating agents before their treatment with idelalisib. Therefore, these patients do not reflect the current population of CLL patients. Due to novel, promising drugs that are introduced in the treatment of CLL recently, such as non-covalent BTK-inhibitors or bispecific antibodies, the value of idelalisib salvage treatment will remain limited to few patients.
